# Global trends in research on eating behaviors among overweight/obese children and adolescents: a bibliometric study from 2003 to 2023

**DOI:** 10.3389/fnut.2025.1494920

**Published:** 2025-04-02

**Authors:** Jiuyuan Wang, Wenjing Huang, Jiaqi Sun, Saiqiong Yin, Jiayi Lin, Pingan Liu, Guixiang Sun

**Affiliations:** ^1^College of Chinese Medicine, Hunan University of Chinese Medicine, Changsha, China; ^2^Administration Department, Hunan Academy Of Chinese Medicine, Changsha, China

**Keywords:** overweight and obese, children and adolescent, eating behaviors, bibliometric, CiteSpace, research focus

## Abstract

**Background:**

Despite the widespread interest in overweight/obese children and adolescents, there is a lack of bibliometric research on the factors influencing eating behaviors.

**Methods:**

Collated and screened research papers published between 2003 and 2023 on eating behaviors in children and adolescents affected by overweight or obesity, searched on January 1, 2024. The primary data, comprising complete records and referenced citations of publications, was extracted from the Web of Science Core Collection. Analysis of data using Bibliometrix of R package, CiteSpace, and VOSviewer.

**Results:**

A total of 2,142 articles were included. The United States had the most publications in the field and was also the center point for world collaborations. Harvard University had the most affiliated publications, while Luis Moreno was the most prolific author. NUTRIENTS was the most published journal. High-frequency keywords included Children, overweight, physical activity, body mass index, and childhood obesity. Research trends include Epidemiology and Environment factors of obesity; Health risks associated with childhood obesity; Key eating habits and interventions for childhood obesity; Prevention and treatment of childhood and adolescent obesity.

**Conclusion:**

This research provides a comprehensive overview of global trends and key areas in studying dietary behaviors among overweight/obese children and adolescents. It offers a detailed summary of recent advancements, emphasizing this field’s critical principles and practices. By exploring these developments, the study highlights the growing importance of this research within global healthcare and suggests pathways for future research and applications.

## 1 Introduction

Over the last several decades, children and adolescent with obesity has become a point of growing concern across the globe, particularly in high-income countries. For instance, the obesity rates among youngsters in the United States soared from 17.7% in 2011–2012 to 21.5 in 2017–2020 (1). More alarming, however, is the fact that obesity is not solely confined to affluent nations. The prevalence of obesity in children and adolescents is continually rising in lower and middle-income countries, too (2). Specifically, in China, the incidence of overweight and obesity among individuals aged 7 to 18 has escalated to an alarming 23.4% (3). The advent of the COVID-19 outbreak in 2019 appears to have further exacerbated the global obesity crisis (4). The outbreak blockade has altered the lifestyle habits of children and adolescents, potentially leading to increased food intake and sedentary behavior (5). It is predicted that the global overweight and obesity rate among children and adolescents could climb to 30% by 2030 (6). Significantly, the majority of teenagers who struggle with obesity tend to carry the condition into adulthood. This progression amplifies their chances of developing obesity-induced complications such as type 2 diabetes, cardiovascular diseases, and asthma (7). Furthermore, obesity can also harm their emotional and social well-being (8). Consequently, targeting and preventing obesity in youngsters has emerged as of global significance.

The causes of obesity are simultaneously influenced by a variety of factors, among which dietary behaviors are considered to be one of the significant influences on overweight and obesity in children and adolescents. Poor eating habits, such as diets high in calories, fat, and sugar, high carbohydrate intake (9), and excessive consumption of sugary beverages (10), lead to excess energy intake over consumption, which in turn triggers weight gain. The problem is compounded by a need for nutritional knowledge and the influence of the social environment (e.g., fast food culture). Many children and their parents have significant gaps in their understanding of healthy eating and nutritional intake, impacting their ability to develop a balanced and well-rounded diet (11, 12). At the same time, changes in the social environment also have an impact on obesity status; for example, the convenience of fast food outlets has been identified as a significant risk factor for childhood obesity (13, 14). The combination of these diet-related factors exacerbates the complexity of the obesity problem in adolescents. However, given the large number of complex research trends, it becomes a formidable challenge to understand the cutting-edge hotspots of research topics in the field and extract accurate and valuable information effectively. This underscores the need for systematic and objective evaluation methods to navigate the intricate landscape of obesity research and identify the most promising avenues for intervention and prevention.

Bibliometrics is recognized as a pivotal tool in scientific research (15). This method not only maps the current state of research to reveal the context of scientific research (16), but also guides clinical practitioners and researchers by analyzing citation patterns and clusters of subject-specific articles, identifying thematic hotspots, and uncovering emerging trends (17).

Bibliometrics is also widely used in the field of obesity research. In Table 1, we list studies related to obesity and diet that are similar to the research topic of this paper. These topics cover obese children, childhood nutrition, pediatric obesity, obesity interventions, and physical activity. However, while existing studies have shed light on various aspects of obesity research, there remains a gap in the bibliometric analysis of the eating behaviors of overweight and obese children and adolescents. This paper addresses that gap by combining several bibliometric tools to explore research hotspots, emerging trends, and potential future research directions in this critical area. Unlike previous studies, our research synthesizes a broader range of methods and incorporates them to maximize their strengths. This study, therefore, contributes new insights by presenting a more comprehensive view of the research landscape concerning childhood obesity and related eating behaviors and attempts to synthesize various tools to identify research hotspots and trends, explore future research directions, support evidence-based decision-making, and reveal gaps and new opportunities in research.

**Table 1:** Similar bibliometrics essay details.

Research direction	Database	Software	Research hot topics	Ref.	Common perspective	Unique perspective
Trends in weight loss for obese children and adolescents	WoSCC	Citespace (√)	Assessment of obesity and pathophysiological mechanism,comprehensive intervention, and bariatric surgery	(120)	C, I, A, J, K	Dual-map overlays Reference analysis
Children’s nutrition	WoSCC	Citespace and VOSviewer	Intestinal microflora, food allergy, overweight and obesity	(55, 121)	C, I, A, J, K	References analysis
Pediatric Obesity	WoSCC	Social network analysis and VOSviewer	physical activity, nutrition, diet, and prevention as well as other more specific challenges	(122)	C, I, A, J, K	Subject Categories Funding
Interventions for obesity	WoSCC	VOSviewer and Bibliometrix (√)	Dietary interventions, exercise interventions and pharmacological interventions are the most popular measures	(123)	C, I,	Thematic map
Physical Activity, Sedentary Behavior, and Diet-Related eHealth and mHealth Research	WoSCC	WOS comes with literature data analysis	In contrast to sedentary behavior and/or diet, most papers are about physical activity	(124)	C, I	Research topics
Sedentary time	WoSCC	Derwent Data Analyzer	The hotspots shifted in the past 10 years, and COVID-19 was the most popular topic of sedentary time research	(125)	C, I, A, J, K	

Common boards have been abbreviated for the sake of table esthetics, C, country analysis; I, Institutions analysis; A, Authors analysis; J, Journals analysis; K, keywords analysis.

## 2 Methods

### 2.1 Data sources

The Web of Science (WoS) was selected as the primary database for this research due to its user-friendly interface and comprehensive analytical tools, ensuring the credibility and quality of the papers utilized. This choice is further validated by numerous prior bibliometric studies in the domain of obesity (18–20), which have also relied on WoS for their foundational data. So we choose WOS as the database.

### 2.2 Research strategy

The literature search spanned from 2003 to 2023. In order to ensure methodological rigor and data validity in this study, we implemented a meticulous literature search and screening strategy over 2 days from January 1, 2024. This constraint was implemented to avert the potential influence of subsequent data updates on the study’s outcomes.

Our search strategy was based on relevant keywords identified through internal discussions and cross-referenced with high-impact meta-analyses. After refining the strategy through testing on the Web of Science Core Collection (WoSCC) database, we finalized the search string used in our study: (TS = (“Body Mass Index” OR “Body Weight Maintenance” OR “Weight Loss” OR “fat” OR “obese” OR “weight maintenance” OR “obesity” OR “loss, weight” OR “lose weight “OR “losing weight” OR “weight reducing” OR “reduce weight “OR “anti-obesity”)) AND TS = (“child” OR “adolescent “OR “teen” OR “youth” OR “juvenile” OR “preteens” OR “preadolescent”) AND TS = (“food intake” OR “sugar intake” OR “sugar consume” OR “fat intake” OR “fat consume “OR “sugar sweetened” OR “sugarsweetened” OR “fizzy drink “OR “snack” OR “fruit” OR “vegetable” OR “fast food” OR “fast-food” OR “food habit” OR “dietary habit” OR “eating habit” OR “eating behavior” OR “diet” OR “nutrition” OR “carbonated beverage” OR “calorie” OR “food habit” OR “dietary habit”). This search yielded a total of 18,990 articles.

**Figure 1:**
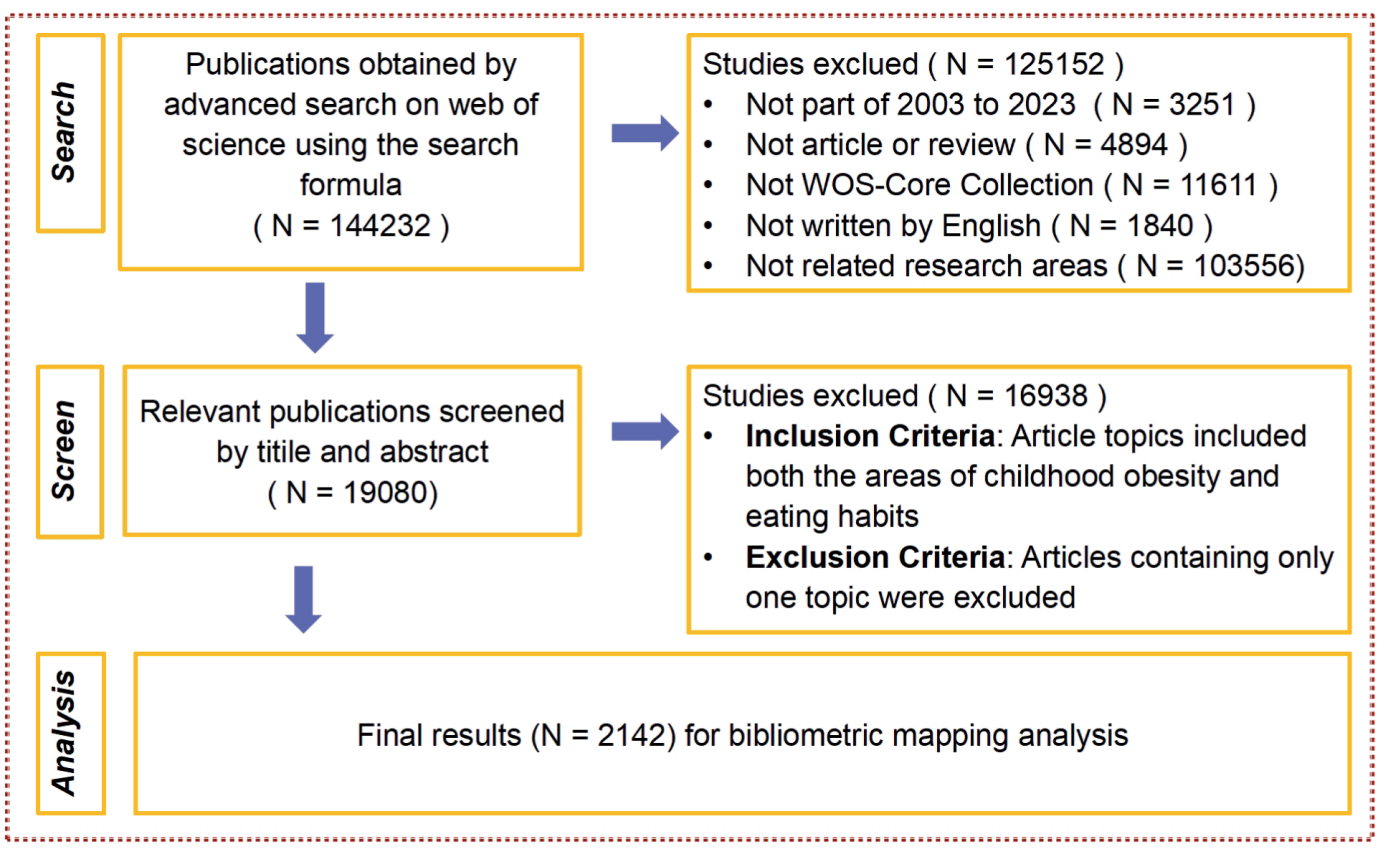
Article screening flow chart.

These articles underwent an initial screening according to predefined criteria, excluding those outside the inclusion requisites. Subsequently, irrelevant articles, determined by examining the title and abstract of each piece, were discarded. This screening process involved four reviewers operating independently. In instances of discrepancy between the reviewers, the final decision was deferred to the lead author. A fifth reviewer conducted final quality control, inspectively appraising the data selected by the initial four reviewers to ensure reliability. The rigorous process outlined above resulted in a corpus of 2,142 articles identified as relevant to our study on the diet behaviors influencing overweight and obese adolescents. The search process is illustrated in Figure 1.

### 2.3 Data analysis

This study employs three respected scientific analytical tools: Citespace 6.25R Advanced, VOSviewer, and the Bibliometrix plugin on R 4.3.2. Their usage is instrumental in conducting a detailed examination and interpretation of the data set.

Citespace, a potent tool for scientometric analysis and data visualization, excels in managing intricate data. It makes scrutinizing scientific and technological literature and promptly identifies and visually portrays knowledge trends and hotspots in the chosen research field (21).

VOSviewer, a software specifically designed for constructing and visualizing bibliometric maps, is employed next. Its unparalleled ability to clearly present large bibliometric maps substantially assists researchers in conducting exhaustive analyses of voluminous data (22).

Lastly, the study incorporates the Bibliometrix plugin for R, an open-source software. This tool provides a well-structured analysis of considerable data, offering possibilities to recognize trends, identify research themes, detect varying disciplinary boundaries, and discover preeminent scholars and institutions (23).

The amalgamation of these three tools, chosen for their robust and efficient capabilities in examining and extracting large complex data sets, furnishes an all-encompassing understanding of prevalent trends and research hotspots.

## 3 Results

### 3.1 Trends in publications and citations

#### 3.1.1 Number of publications

**Figure 2:**
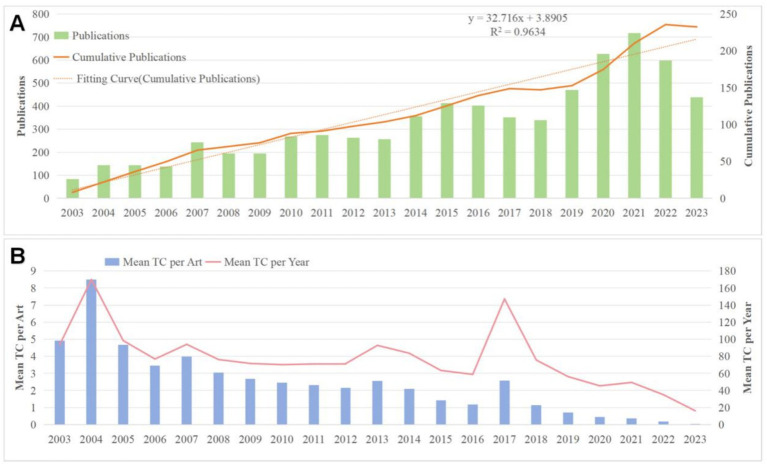
**(A)** Cumulative chart of annual publications. **(B)** Mean TC per article and year chart.

The number of publications over time can serve as an insightful indicator of research trends and advancements in a specific field. From the WoSCC database, we included 2,142 articles published between 2003 and 2023, as shown in Figure 2A. From 2003 to 2007, the field saw an initial surge in scholarly interest. The number of published papers grew from 26 in 2003 to 76 in 2007, marking a growth rate of 76.7%. This considerable increase not only signifies the academic establishment of the field but also highlights its potential for significant research impact and attraction. Beginning in 2008, the number of publications stabilized between 60 and 110 annually, indicating sustained and steady growth. This period demonstrates the field’s enduring appeal and evolving research dynamics. Between 2017 and 2020, despite fluctuations where publication numbers briefly declined and rebounded, the output remained robust, consistently exceeding 100 papers annually. The peak of 196 publications in 2020 likely reflects the influence of specific pivotal events or emerging research hotspots that catalyzed scholarly activity. By 2021, the publication count reached an impressive total of 224, marking an astonishing increase of 761.5% compared to 2003. This figure not only quantitatively showcases the prolific output of research in the field but also serves as robust evidence of its profound academic influence and depth of inquiry.

#### 3.1.2 Citations of publications

These papers have been cited a total of 72,637 times. We extracted two metrics, Mean TC per Art and Mean TCper Art, using the Bbibliometrix plugin of R, in Figures 2A, B. Mean TC per Art refers to the average Number of citations per article. If the value is high, it usually means that the study has attracted extensive attention and citations from other scholars. Figure 2B shows that Mean TC per Art peaked in 2004 and was high from 2003 to 2007, after which it declined.

Mean TC per Year is the average Number of citations per unit of time (year). This metric can be used to compare citations for different years of research or fields of study and is more indicative of trends in the popularity of a particular research topic or field of study. Then, it may indicate that the research in that field or topic is getting more and more attention and citations from peers. As can be seen in Figure 2B, also in 2004, the value peaked and then gradually declined, with a second growth peak from 2016 to 2017, followed by a gradual decline.

### 3.2 Trends in national publications and cooperation

Eating behaviors of overweight and obese children and adolescents are a globally recognized research priority. A total of 105 countries contributed to research on eating behaviors in overweight and obese children and adolescents. The top 10 countries in terms of publications are listed in Table 2. Ranked first in the total Publications (TP) of publications is the United States (TP = 821, H = 96), which also holds the leading position considering Total Citations (TC) and H-index. The following positions are filled by Australia (TP = 180, H = 43), the United Kingdom (TP = 178, H = 46), China (TP = 143, H = 28), and Spain (TP = 117, H = 30). Among these contributions, the collective output of the top three countries accounted for over 50% of the research findings, underscoring their substantial engagement and pivotal role in advancing knowledge within this domain.

**Table 2:** Top 10 countries/regions in terms of publications.

Rank	Countries/Regions	TP	TC	H-index
1	USA	821	39,846	96
2	Australia	180	9,281	43
3	United Kingdom	178	9,869	46
4	China	143	5,238	28
5	Spain	117	6,073	30
6	Canada	107	6,806	31
7	Brazil	97	4,315	23
8	Germany	83	5,855	31
9	Netherlands	75	6,026	29
10	Italy	62	4,543	21

**Figure 3:**
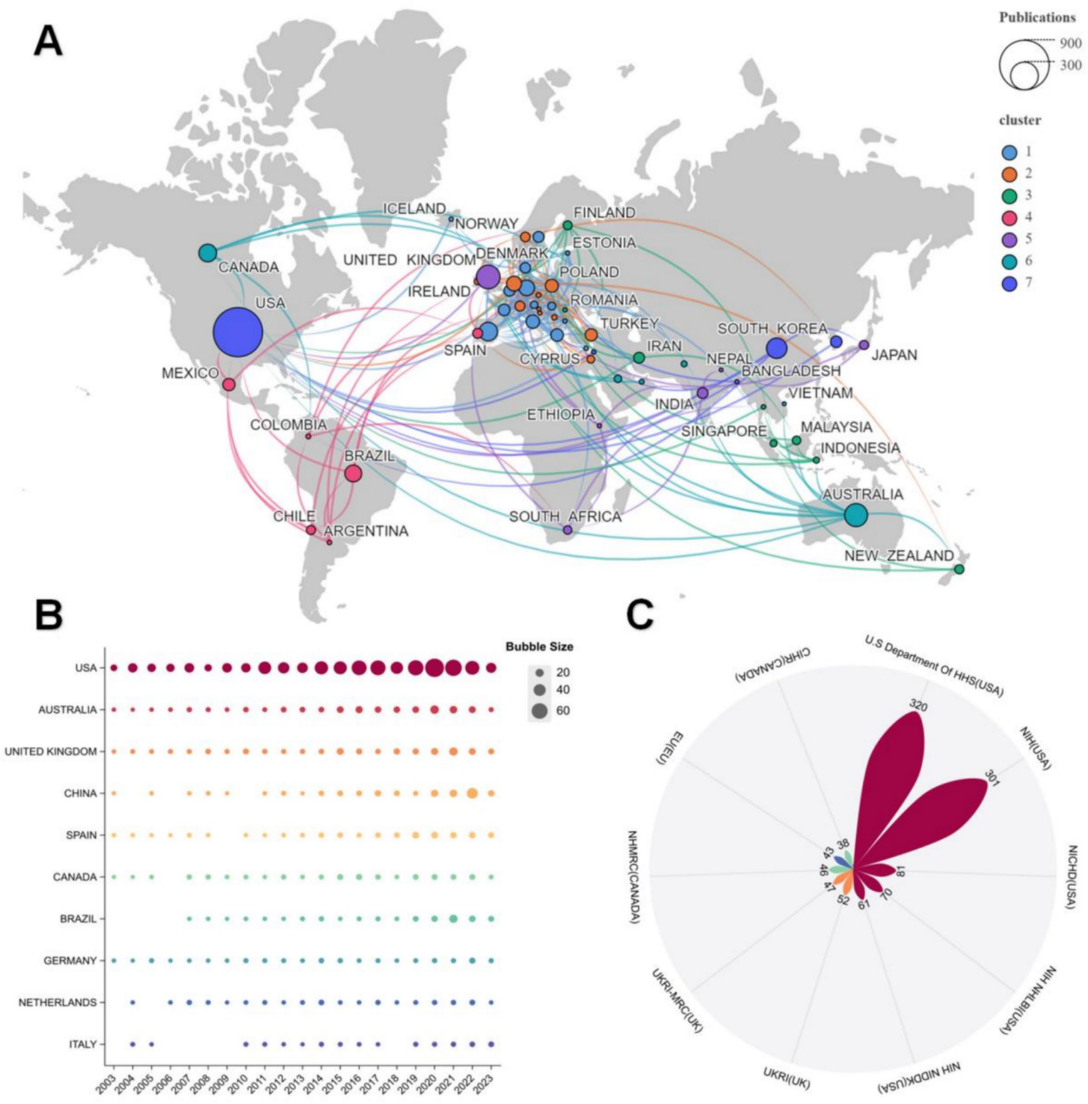
**(A)** Country cooperation networks. Lines indicate partnerships, colors indicate different clusters and node size indicates the number of publications. **(B)** Annual production bubble chart in top 10 countries. **(C)** Petal chart of top 10 funding sources.

Employing data culled from VOSviewer allowed us to craft a visualization of the collaborative relationships between countries in Figure 3A. These relationships form seven distinct clusters, within which the United States having the most publications shares robust ties with several Western European nations, Australia, and South Asian countries (including India and Singapore). In contrast, the United Kingdom exhibits a more decentralized collaboration framework, boasting partnerships with South Africa, New Zealand, and various other countries.

In order to analyze the scholarly output of the top 10 countries in this field more effectively, we extracted data from WOS and used it to create an annual publication bubble chart, as shown in Figure 3B. Notably, the United States, Australia, the United Kingdom, and Germany have all sustained consistent research output in this field over the past two decades. The publications of the USA displayed accelerated growth from 2018 to 2020. Although faced with intermittent breaks, all countries recorded increases over the past 3 years.

Figure 3C enumerates this field’s top 10 funding sources according to the quantity of funded research. With 320 funded projects, the US Department of Health and Human Services (HHS) takes the top spot, followed by the National Institutes of Health (NIH) with 301, the National Institute of Child Health and Human Development (NICHD) with 81, The National Heart, Lung, and Blood Institute (NIH NHLBI) with 70, and the National Institute of Diabetes and Digestive and Kidney Diseases (NIH NIDDK) with 61. Consequently, the top five funding sources originate from the United States and are governmental agencies.

### 3.3 Trends in institutional publications and cooperation

A total of 2,667 institutions contributed to research in the field. Setting the number of publications at 20, 20 institutions met this criterion, and we refer to these as “active producers.” Table 3 enumerates the top 10 institutions in terms of publication numbers. With 79 publications, Harvard University is the leading institution, standing out not just by the TP but also by its Total Citations (TC) and H-index. Following closely are the University of North Carolina (*n* = 71), the University of California System (n = 65), UNIVERSITY OF NORTH CAROLINA CHAPEL HILL (*n* = 54), and the University of Minnesota System (n = 50). Notably, all these institutions are based in the United States.

**Table 3:** Top 10 institutions in terms of publications.

Rank	Institution	TP	TC	H-index	Country
1	Harvard University	79	7,855	28	USA
2	University of North Carolina	71	4,272	27	USA
3	University of California System	65	5,284	25	USA
4	University of North Carolina Chapel hill	54	4,095	27	USA
5	University of Minnesota System	50	3,319	29	USA
6	University of Sydney	50	1,427	19	Australia
7	University of London	48	5,246	25	UK
8	Harvard T H Chan School of Public Health	47	5,689	22	UK
9	Deakin University	39	2,330	19	Australia
10	Ciber Centro De Inverstigacion Biomedica En Red	37	3,739	15	Spain

**Figure 4:**
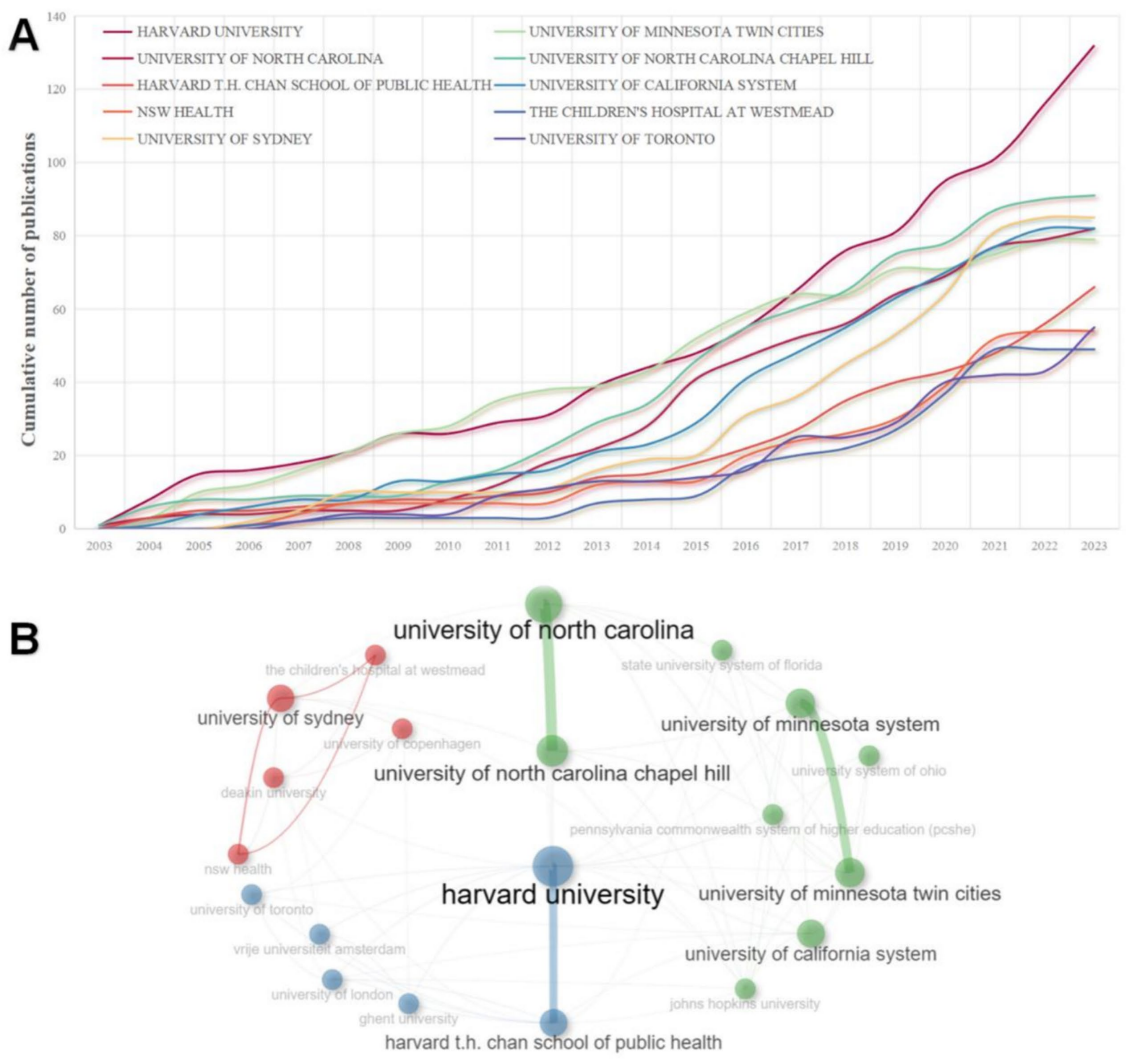
**(A)** Top 10 cumulative graph of publications of large institutions. **(B)** Institutional cooperation network. The size of the node indicates how many publications are made, the thickness of the line indicates the strength of the collaboration, and the color indicates clustering.

A detailed chronological publication analysis of these top 10 institutions is illustrated in Figure 4A. Notably, research in this domain by Harvard University, the University of North Carolina, the UNIVERSITY OF NORTH CAROLINA CHAPEL HILL, and the University of Minnesota System started in 2003 and has persisted since then. A significant rise in the Number of publications was observed across all institutions between 2009 and 2014, with Harvard University and the University of California System showing the most prominent increases. This growth continued from 2015 to 2023, during which all institutions dramatically escalated their publication numbers.

Figure 4B visualizes the collaboration among these active producers using the Bibliometrix plugin for R and generating a collaboration map. Three distinct clusters (green, blue, and red) emerge, indicating separate collaborative networks. Within the green cluster, the University of North Carolina and the University of North Carolina Chapel Hill exhibit extensive collaborations and are the most significant nodes. In the blue cluster, Harvard University is the most significant node and collaborative within the network, mainly with its branch. Lastly, the red cluster’s largest node is the University of Sydney, which displays a triangular collaboration with the Children’s Hospital at Westmead and NSW Health.

### 3.4 Trends in author publications and cooperation

A total of 9,580 authors contributed to this field. Table 4 lists the top 10 authors in terms of the Number of publications. Luis Moreno from Spain (TP = 29, H = 16) is ranked first, Barry Popkin (TP = 22, H = 18) is ranked second but has the highest H-index of all authors at 18 and also has a TC of 2,871, Louise Baur (TP = 19, H = 13) is ranked third, Sarah P Garnett (TP = 17, H = 11) and Yannis Manios (TP = 16, H = 9) followed.

**Table 4:** Top 10 authors in terms of publications.

Rank	Name	TP	TC	H-index	Country
1	Luis Moreno	29	1,151	16	Spain
2	Barry Popkin	22	2,871	18	USA
3	Louise Baur	19	861	13	Australia
4	Sarah P Garnett	17	645	11	Australia
5	Yannis Manios	16	425	9	Belgium
6	Kersting Mathilde	14	590	10	Germany
7	Dianne Neumark-Sztainer	13	546	11	USA
8	David S Ludwig	12	2,897	10	Denmark
9	Cara B Ebbeling	12	2,929	10	Denmark
10	Wang Youfa	11	488	8	China

**Figure 5:**
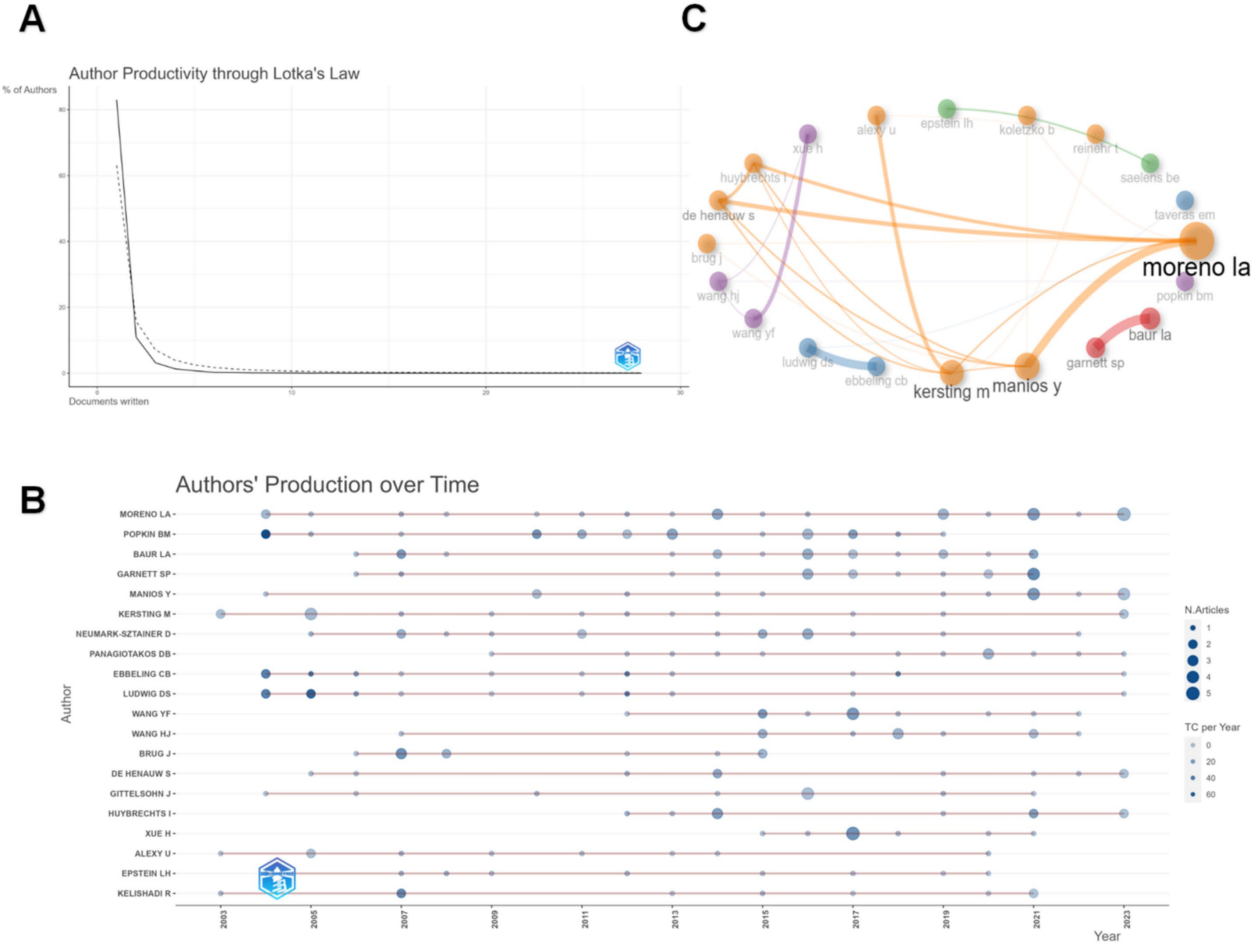
**(A)** Lotka’s law. **(B)** Authors’s production over time chart. The size of the node indicates the number of publications and the shade of the color indicates the number of times it has been cited. **(C)** Author cooperation network. The size of the node indicates how many publications are made, the thickness of the line indicates the strength of the collaboration, and the color indicates clustering.

We analyzed authors in the field using Lotka’s law from the bibliometrix in Figure 5A. Among the 9,580 authors, the group that has published one paper is the largest, accounting for 83% of the total. As the number of publications per author increases, the number of contributors at each level decreases sharply. Particularly notable is the sharp contraction in the number of authors who have published four or more papers. This trend reveals a general phenomenon: the vast majority of authors in the field have limited output, while a few highly productive authors contribute a significant proportion of the scholarship. This distribution emphasizes the centrality of high-producing authors in creating academic knowledge and reflects the uneven distribution of academic output.

After setting the minimum Number of publications to 8 in VOSviewer, 20 active producing authors were extracted. In Figure 5B, we present the annual production of active producing authors. The dark blue color in the figure indicates the influential articles in that year; Popkin and Ebbeling all made influential studies in 2004. Moreno, the top article producer, has been broadly and consistently active in the field since 2004. In addition, many authors have also produced high-impact literature in 2021.

In addition, we produced the collaborations of the active producing authors in Figure 5C. These authors form five color clustering themes (orange, purple, blue, red, and green). Moreno strongly collaborates with Manios and Kersting, ranked fifth and sixth in the Number of publications. The blue clustering has the same two scholars from Denmark as the main collaborators. The green and red clusters both use only one layer of collaboration. Popkin, who ranks second in the TP, is in the purple clustering, with fewer collaborations with other nodes, and the other three authors of this cluster are all from China.

### 3.5 Trends and thematic changes in journal publications

The publications were published in 423 journals. Table 5 lists the top 10 journals in terms of Number of publications. NUTRIENTS are ranked first (*n* = 175), followed by PUBLIC HEALTH NUTRITION (*n* = 64), PEDIATRIC OBESITY (*n* = 61), INTERNATIONAL JOURNAL OF OBESITY (*n* = 54), and BMC PUBLIC HEALTH (*n* = 50). Although INTERNATIONAL JOURNAL OF OBESITY does not have as many publications as the first three journals, its TC and H-index values are ranked first, which shows that the articles it publishes are of very high quality and have a high impact on peers. Looking at the Quartile of the impact factor, there is a significant difference in the level of these 10 journals, ranging from Q1 to Q3. However, most of the journals are still in Q1 and Q2, indicating that the research in this field is generally of high quality.

**Table 5:** Top 10 journals in terms of publications.

Rank	Journal	TP	TC	H-index	IF
1	NUTRIENTS	175	1904	23	Q1/5.9
2	PUBLIC HEALTH NUTRITION	64	1,650	22	Q3/3.2
3	PEDIATRIC OBESITY	61	1,222	19	Q1/3.8
4	INTERNATIONAL JOURNAL OF OBESITY	54	4,356	31	Q2/4.9
5	BMC PUBLIC HEALTH	50	1,391	19	Q2/2.5
6	OBESITY REVIEWS	44	2,411	23	Q1/7.3
7	OBESITY	40	1,588	22	Q1/3.7
8	BRITISH JOURNAL OF NUTRITION	38	1,241	17	Q3/3.6
9	PLOS ONE	36	576	14	Q2/2.7
10	PROCEEDINGS OF THE NUTRITION SOCIETY	28	58	4	Q1/5.5

**Figure 6:**
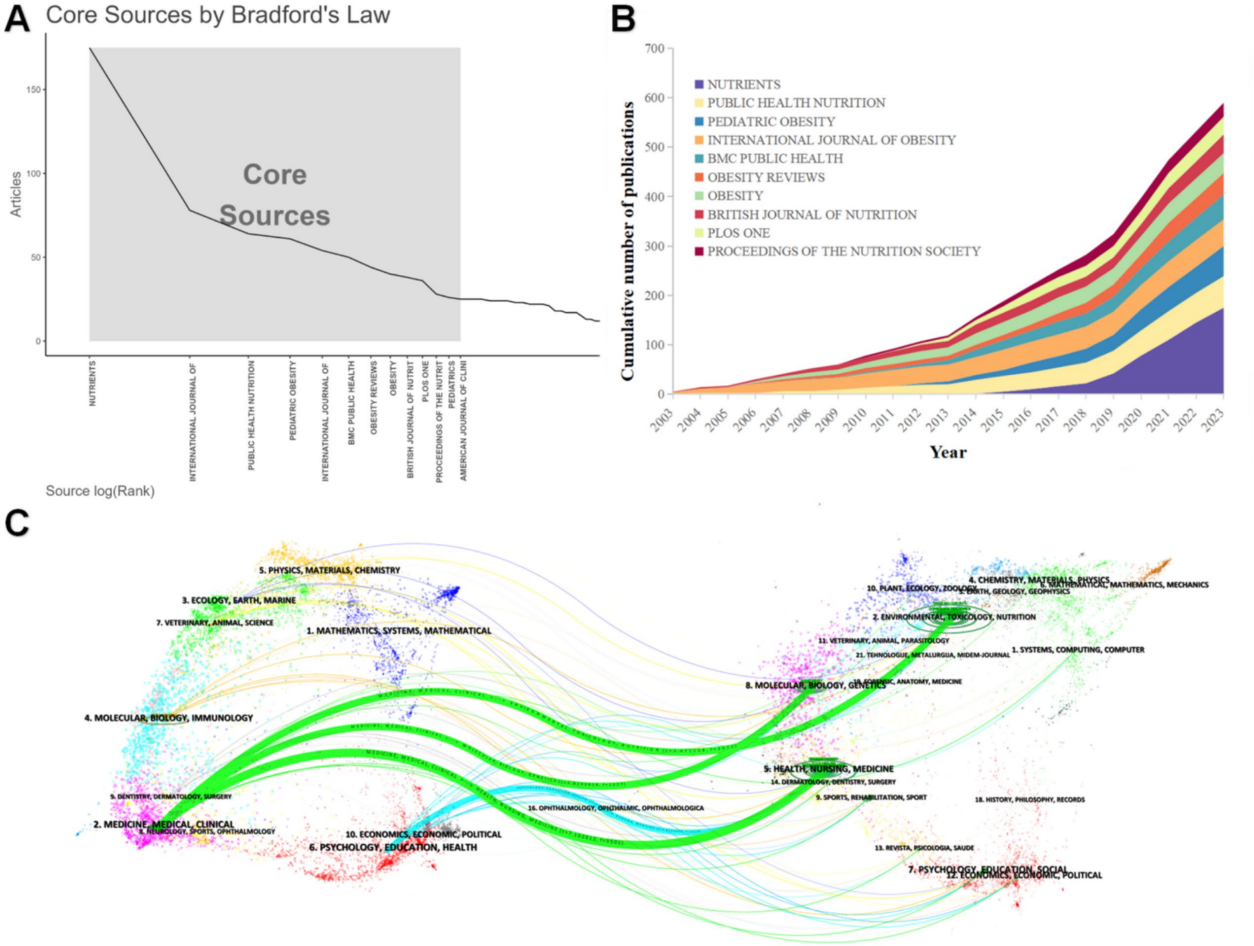
**(A)** Bradford’s law. **(B)** Cumulative publication volume of the top 10 journals charted. **(C)** Journal double map overlay. The left side indicates the citing literature and the right side indicates the cited literature. A line indicates a citation relationship, with the left side pointing to the right side indicating that the article on the left cites the article on the right.

According to Bradford’s Law, the distribution of scientific journal papers follows a specific pattern, and Figure 6A, extracted using the Bibliometrix plugin, clearly shows this trend. The analysis reveals that 13 journals comprise the Core Sources, having published many papers on obesity-related eating behaviors in children and adolescents. Notably, the top-ranked journal, NUTRIENTS, plays a particularly crucial role in this field. Therefore, focusing on these core journals will significantly enhance the efficiency and quality of information retrieval when conducting literature searches and research on related topics. This approach helps researchers quickly locate key literature and ensures a comprehensive and in-depth research foundation.

Figure 6B lists the cumulative annual production of papers for the top 10 journals in terms of publications, and it can be seen that between 2003 and 2007, only the INTERNATIONAL JOURNAL OF OBESITY, PUBLIC HEALTH NUTRITION, BRITISH JOURNAL OF NUTRITION, the Three journals were publishing articles in the field. By 2012, all the journals had started publishing articles in this field. NUTRIENTS started publishing relevant papers in 2010 and, with rapid growth, became the journal with the highest number of articles. There was a clear growth inflection point in 2013, with rapid growth in the Number of articles published in the field.

To further explore the history of articles in this field, we created a double citation map for the journal using Citespace 6.25R Advanced Edition in Figure 6C. A journal double-map overlay is a specialized method for studying citation relationships and thematic evolution among academic journals. Simply put, it involves overlaying two journal citation graphs so one can observe the transmission of a scientific issue from one field to another. The results of the graph show that the number 2 on the left (MEDICINE, MEDICAL, CLINICAL), points to the number 2 on the right (ENVIRONMENTAL, TOXICOLOGY, NUTRITION; *Z* = 2.471158, *f* = 2072), the number 8 on the right (MOLECULAR, BIOLOGY. GENETICS; *z* = 2.829, *f* = 2,337) and #5 on the right (HEALTH, NURSING, MEDICINE; *z* = 7.1026, *f* = 5,501); #6 on the left (PSYCHOLOGY, EDUCATION, HEALTH) points to #5 on the right (HEALTH, NURSING. MEDICINE; *z* = 1.85525, *f* = 1,616).

### 3.6 Reference analysis

The analysis of references helps researchers gain a deeper understanding of the research field’s development and determine the research heat and trend of a specific field or topic, which is very helpful for researchers to locate the research direction and choose the research topic. Therefore, we analyze the references with high LCS scores and co-cited reference.

#### 3.6.1 High LCS references

Local Citation Score (LCS) is a metric that indicates the number of times a document has been cited in a particular field. LCS can help assess the quality and impact of academic articles. Articles with high scores are usually considered to have a higher impact in their field or region, and these papers may be critical works that lead research directions and trends and are essential for understanding the development of the field. We used the Bibliometrix to extract the top 10 cited papers in the field with LCS in Table 6.

**Table 6:** Top 10 journals in terms of publications.

Authors	Title	Journals	LCS
Cole TJ, et al	Establishing a standard definition for child overweight and obesity worldwide: international survey	BMJ	329
Mercedes de Onis, et al	Development of a WHO growth reference for school-aged children and adolescents	Bull World Health Organ	118
Barlow SE, et al	Expert committee recommendations regarding the prevention, assessment, and treatment of child and adolescent overweight and obesity: summary report	PEDIATRICS	114
Ludwig DS, et al	Relation between consumption of sugar-sweetened drinks and childhood obesity: a prospective, observational analysis	LANCET	104
NCD-RisC	Worldwide trends in body-mass index, underweight, overweight, and obesity from 1975 to 2016: a pooled analysis of 2416 population-based measurement studies in 128·9 million children, adolescents, and adults	LANCET	101
Ogden CL, et al	Prevalence of childhood and adult obesity in the United States, 2011–2012	JAMA-J AM MED ASSOC	96
WHITAKER RC, et al	Predicting obesity in young adulthood from childhood and parental obesity	NEW ENGL J MED	94
Ogden CL, et al	Prevalence and trends in overweight among US children and adolescents, 1999–2000	JAMA-J AM MED ASSOC	85
Malik VS, et al	Intake of sugar-sweetened beverages and weight gain: a systematic review	Am J Clin Nutr	84
Ogden CL, et al	Prevalence of obesity and trends in body mass index among US children and adolescents, 1999–2010	JAMA-J AM MED ASSOC	82

The one paper with the top LCS was published in 2000, which investigated the weight of adolescent children in six different countries and developed a more international definition of children with overweight and obesity using body mass index as an indicator (24). A standard was provided for the field so that other researchers and professional organizations could conduct consistent research and practice globally. This development has been a substantial factor in advancing research in this field. The second paper enriches the field of research on overweight and obese children and fills a gap in this area for the World Health Organization by constructing growth curves for children and adolescents between the ages of 5 and 19 years using the Box-Cox power index method (25). In the same year, an expert committee of 15 professional organizations as representatives made comprehensive recommendations for the prevention, assessment, and treatment of overweight and obesity in children and adolescents, including regular assessment, diet, physical activity, and many other aspects (26), which provides practical and comprehensive guidelines for subsequent research. The fourth paper is from the Lancet. This study recruited 548 ethnically diverse American children and conducted a 19-month follow-up survey to explore the relationship between sugary drink consumption and childhood obesity. The results showed that consumption of sugary drinks was associated with childhood obesity (27). These findings have important implications for preventing and treating childhood obesity, provide a scientific basis for changing children’s eating habits, and provide essential information for policymakers.

#### 3.6.2 Hot spots for co-citation references

**Figure 7:**
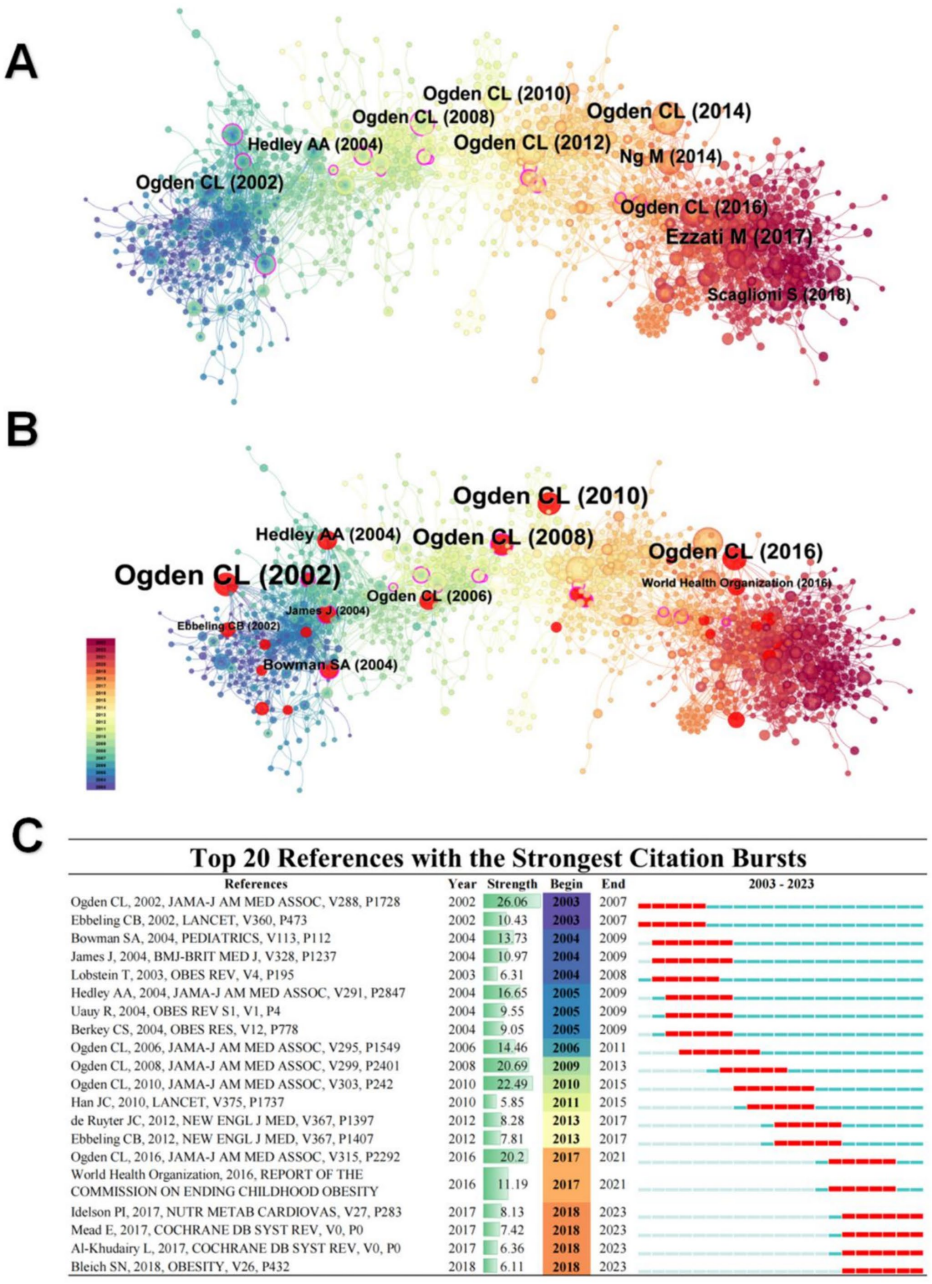
**(A)** Co-citations references network. Node size indicates frequency, color indicates different years. **(B)** Top 10 bursts of co-citations references. Nodes in red indicate burst literature. **(C)** Top 10 burst citations references chart. In the 2003–2023 column, red indicates the year of the burst, and blue indicates the year when the literature began to be cited until it decline.

Co-citation reference is when a particular reference is co-cited by two or more documents at the same time. These documents have some connection with each other, so they are cited together (28). Analyzing the temporal patterns of co-citation reference enables tracing intellectual evolution within a research domain. It elucidates the emergence and decline of topics, revealing the ebb and flow of scholarly attention. Highly co-citation reference signifies pivotal contributions, forming the nucleus of the field’s knowledge base. By scrutinizing these core references, one gains insight into the conceptual underpinnings and trajectory of the discipline. We used the Citespace software to construct a co-citation reference network graph, with Figure 7A highlighting the 10 most frequently cited papers. The publication years of these papers are differentiated by color coding. Notably, six of these papers were authored by Ogden CL and published between 2002 and 2016 in the Journal of the American Medical Association (JAMA), a leading medical journal. Ogden’s research has significantly impacted the fields of medicine and public health, particularly in understanding the developmental dynamics of children and adolescents with obesity. These studies span two distinct periods, 1999–2010 (29–32) and 2011–2014 (33, 34), guiding future research directions in identifying factors influencing childhood and adolescent obesity. Furthermore, Ogden’s work provides essential scientific evidence for preventing and controlling dietary issues among young populations.

#### 3.6.3 Burst analysis of co-citation references

Bursts in the co-citation network represent hot references in the research field at a given time. These “bursts” can help researchers identify essential advances in the field, leading directions, and possible research trends. Figure 7B shows the top 10 papers that exhibit hotspots in the co-citation reference, and Figure 7C lists detailed data for the top 20 papers. Four Ogden CL’s publications are ranked highest in citation strength until 2021, demonstrating a consistently high impact since 2002. However, the focus on these studies has slightly diminished in recent years, with Ogden’s most recent population studies available up to 2014. Notably, two papers that gained increased attention after 2021, up to 2023, concentrated on the effects of eating habits and behaviors on the health of children and adolescents (35, 36). These findings are pivotal for understanding and addressing issues related to overweight and obesity among adolescents.

### 3.7 Keyword trends

**Figure 8:**
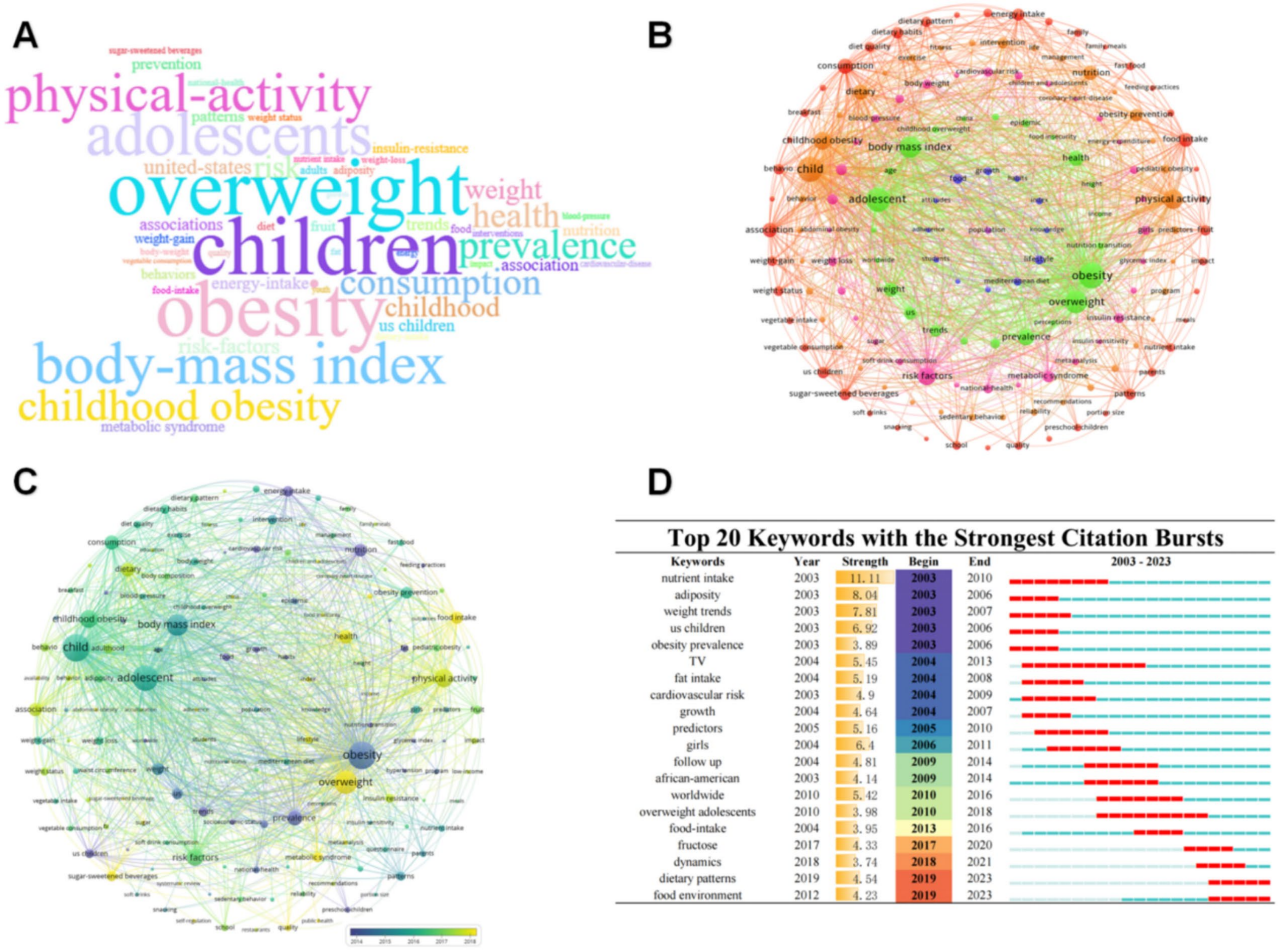
**(A)** WordCloud. The size of the word indicates the frequency of occurrence; the larger the word, the more frequently it occurs. **(B)** Keyword co-occurrence map. The node size indicates how often the keyword appears, the larger the higher the frequency. Node color indicates different clusters. Lines indicate that two keywords co-occur in the same paper or papers. **(C)** Keyword temporal overlay map. Different colors of the nodes indicate different years, the rest is consistent with **(B)**. **(D)** Keyword burst chart.

A total of 2,742 keywords were extracted in this domain. The keywords with relatively high frequency in the domain are listed in Figure 8A, such as children, overweight, physical activity, body mass index, childhood obesity, prevalence, and consumption of adolescents. In addition, we extracted co-occurring keywords and outbreak keywords.

#### 3.7.1 Keyword co-occurrence map

Keyword co-occurrence analysis refers to the simultaneous occurrence of two or more keywords in the same research document. By constructing a co-occurrence keyword map we can see the distribution of keywords in the research field the popularity trend of topics and the relationship between topics. We used VOSviewer to extract 150 words to make a keyword co-occurrence map see Figure 8B. It can be seen that these keywords form 5 clusters of different colors (there is only one keyword in the most central position which is not put into the discussion). The first red color includes food intake, fast food, energy intake, dietary pattern, dietary habits, sugar-sweetened beverages, vegetable consumption, nutrient intake. These keywords highlight the significant relationship that exists between the ingestion of nutrients and dietary customs

The second orange cluster includes childhood obesity, intervention, nutrition, pediatric obesity, physical activity, fitness, and management. Topics relate to obesity prevention, exercise and fitness, and lifestyle interventions. This includes the relevance of exercise interventions, lifestyle management, childhood obesity prevention methods, and physical activity.

The third pink cluster includes cardiovascular risk, coronary heart disease, energy expenditure, insulin resistance, metabolic syndrome, risk factors, and blood- pressure, focusing on physical health indicators, and the cardiovascular risk of obesity, which emphasizing the link between obesity and, excessive sugar intake and long-term health risks.

The fourth green cluster, including adolescent, obesity, overweight, prevalence, food insecurity, epidemic, trends, and nutrition transition, focuses on epidemiologic and socioeconomic factors of obesity.

The fifth cluster, identified by the color blue, encompasses themes such as growth, habits, knowledge, lifestyle, adherence to Mediterranean diet, attitudes, and food. It primarily focuses on the interrelations between diet, growth, lifestyle, and dietary knowledge.

In addition, the temporal trend of the development of these keywords is shown in Figure 8C, where it can be seen that in the early years (around 2014), the research hotspots were in prevalence, trends, US children, food, habits, growth, after that to the period between 2016 and 2017, dietary pattern, dietary habits, risk factors, fast food, obesity prevention, consumption, are more popular. After 2018, overweight, food intake, health, sugar-sweetened beverages, and metabolic syndrome became popular.

#### 3.7.2 Co-citation burst keywords

“Burst keywords” pertain to terms that occur multiple times within a specified period and reflect emerging research themes or trends in a field of study that are receiving rapid attention. We employed Citespace to generate these “burst keywords,” as depicted in Figure 8D. In this field, the hot keywords from 2003 to 2010 are nutrient intake, adiposity, weight trends, us children, obesity prevalence, fat intake, cardiovascular risk, growth, and predictors, of which nutrient intake has the highest strength (Strength = 11.11). Between 2011 and 2020, the hot keywords are girls, follow-up, TV, African-American, worldwide, worldwide, overweight adolescents, food-intake, fructose, and girls having the highest strength (Strength = 6.4). Lastly, the keywords for the last 3 years, from 2021 to the present, include dynamics, dietary patterns, and food environment, with dietary patterns (Strength = 4.54) having the highest strength.

### 3.8 Analysis of co-citation networks

**Figure 9:**
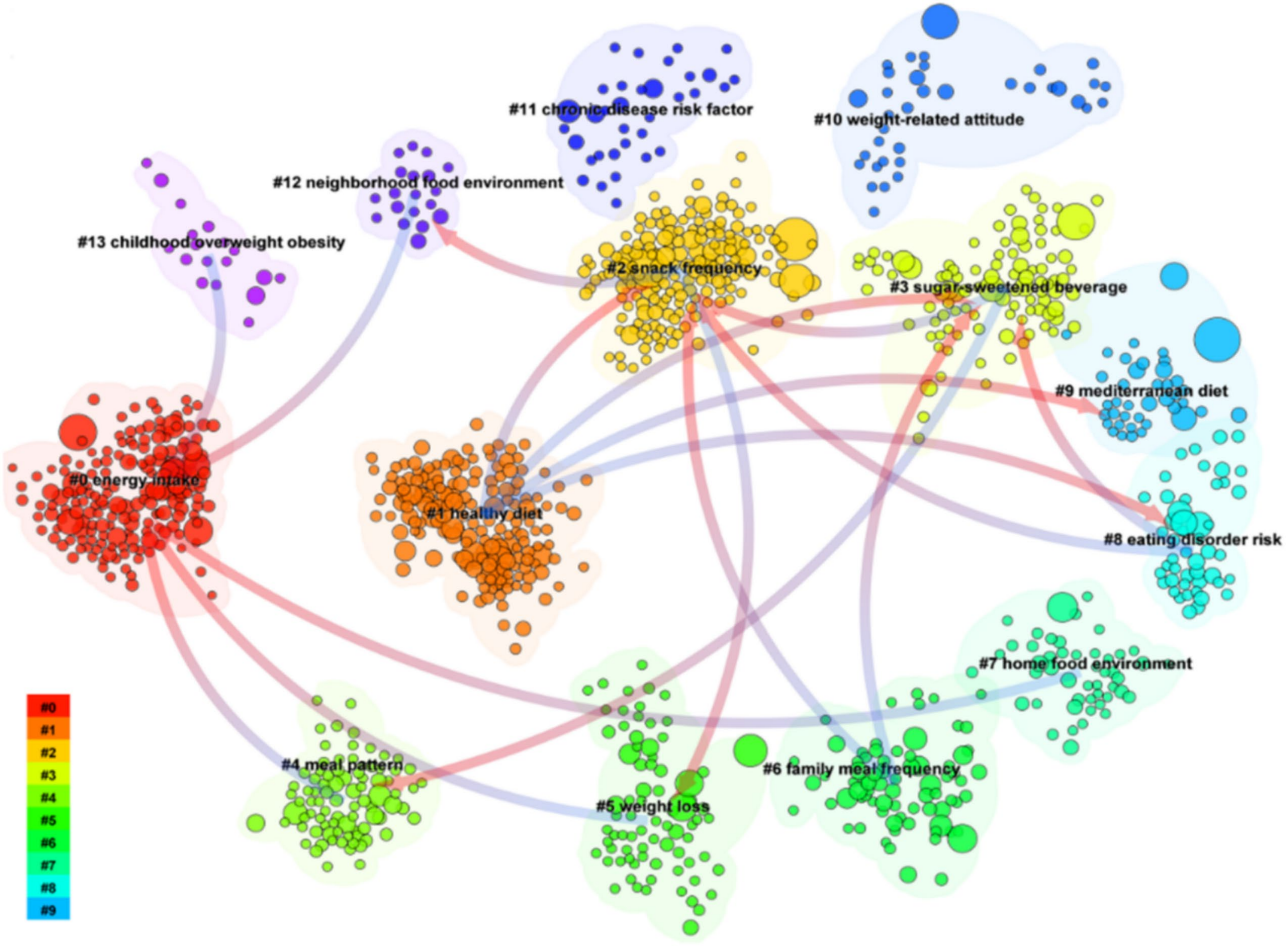
Co-citation network map. Node colors indicate different years. The lines indicate the relationship between the different clusters, with A pointing to B, meaning that B is the research base for A.

Citespace’s unique feature, reference co-citation networks, mainly helps identify core themes in related literature and provides an in-depth understanding of a research area or a specific issue. It can clarify the field’s current position and development trend and provide strategies and directional references for future research. At the same time, it reveals the intersection and connection between different disciplines or fields. The reference co-citation networks were mapped from the title of cited references using the log-likelihood algorithm (LLR), and 14 clusters extracted from the references of 2,142 cited literature were thoroughly examined, In Figure 9. This literature formed a total of 14 clusters, which were #0energy intake, #1 healthy diet, #2snack frequency, #3 sugar-sweetened beverage, # 4meal pattern, #5 weight loss, #6 family meal frequency, #7 home food environment, #8 eating disorder risk, #9 Mediterranean diet, #10 weight-related attitude, #11 chronic disease risk factor, #12 neighborhood food environment, #13 childhood overweight obesity. A classification pattern similar to the keyword co-occurrence map can be observed from the obtained data. In addition, the straight lines for each cluster in the figure show their developmental relationship. It can be seen that there are intricate relationships among multiple clusters, specifically #12, #13, #4, #5, #7 pointing to #0; #6, #8, #3 pointing to #2; #1, #6, #8 pointing to #3, #3 pointing to #4, #2 pointing to #5; #1 pointing to #1 respectively; #2 pointing to #5; #2 pointing to #5; and #1 pointing to #1, respectively. 2 points to #5; #1 points to #8 and #9 respectively; #2 points to #12. These relationships show that energy intake, healthy diet, snack frequency, and sugar-sweetened beverages are the foundational research bases in this field. These core themes have gradually evolved into more complex topics, including meal patterns, home food environment, weight loss, neighborhood food environment, and the Mediterranean diet. This progression highlights a shift from essential dietary components to a more comprehensive focus on lifestyle and environmental factors influencing childhood obesity.

## 4 Discussion

In the present study, we harnessed cutting-edge bibliometric tools, including Citespace, Vosviewer, and Bibliometrics, to comprehensively explore the literature about the eating behaviors of overweight/obese children and adolescents. Our investigation extended beyond mere publication counts to encompass a meticulous quantitative analysis of pivotal components such as the nations, research institutions, leading authors, prominent journals, and salient keywords that have shaped the discourse within this domain. The analysis covers 2,142 articles published over 20 years from 2003 to 2023, with a cumulative citation count of 75,409, and the number of publications is increasing each year, indicating an upward trend in the field. Researchers from 105 countries/territories around the globe participated in the study of eating behaviors of overweight and obese adolescent children, demonstrating the global significance and relevance of this topic to global public health.

### 4.1 Main findings

Among the major countries involved in research on the eating behaviors of overweight and obese adolescent children, the United States is a notable leader in both the number of publications and the impact of articles. This prominence is closely related to the rising rates of childhood obesity and the consistent and steady funding available in the United States. Since 2003, US research activities have not only flourished domestically but have also fueled international collaborations and research trends in the field through robust funding support (Figures 3A–C). Grants from the US Department of Health and Human Services (HHS) and several National Institutes of Health (NIH)-affiliated institutes, including the National Institute of Child Health and Human Development (NICHD), the National Heart, Lung, and Blood Institute (NHLBI), and the National Institute of Diabetes and Digestive and Kidney Diseases (NIDDK), demonstrate a deep understanding of adolescent obesity and a focus on health determinants in their funding allocations.

US funding supports fundamental research and fosters interdisciplinary and cross-border collaborations. As illustrated in Figure 3A, the United States sits at the center of a network of international collaborations, maintaining strong partnerships with research institutions worldwide. This collaborative model integrates research resources from various countries and regions to explore the complex associations between dietary behaviors and obesity and the underlying social, economic, and environmental determinants of health. Meanwhile, Australia, the United Kingdom, and China have invested substantially in this field (Figure 3C). Their respective funding agencies, such as the National Health and Medical Research Council (NHMRC) in Australia, the United Kingdom’s Agency for Research and Innovation (UKRI) and its subsidiary, the Medical Research Council (MRC), and China’s National Natural Science Foundation, support both national and international research programs. These efforts have provided platforms for researchers to explore eating behavior change strategies, nutrition education interventions, and the influence of family and social environments on adolescents’ eating habits.

Consequently, these initiatives contribute to developing more effective prevention and intervention measures for adolescent overweight and obesity. Thus, the U.S. leadership in adolescent eating behavior research is not only due to the continuity and stability of its financial support but also reflects a deep understanding of and active response to the country’s rising childhood obesity rates. This strategy of combining practical problem-solving with substantial research funding provides valuable experience and insights for addressing adolescent obesity on a global scale.

Most of the top 10 research institutions with the most publications in this field are universities and research institutes in the United States, including Harvard University, the University of North Carolina, and the University of California System. These findings coincide with previous analyses related to countries, further exemplifying the prominent role of U.S. research activities in this specific area of study. Harvard University leads the way with 79 publications and 7,855 citations, recognizing its research’s quality and significant contribution to the field. The University of Minnesota System has the highest H-index. These are the premier overweight and obesity research institutions in the United States and have significantly contributed to studying children and adolescents and eating behaviors.

Among the top 10 authors, Luis Moreno is the foremost contributor in terms of publication volume, with Barry Popkin and Louise Baur closely following, underscoring their exceptional dedication to advancing knowledge in this domain. Since 2004, Professor Luis Moreno has been at the forefront of identifying the profound effects of dietary environmental shifts on the eating behaviors of children and adolescents, advocating for a meticulous examination of these behaviors (37). Over the subsequent decade, his research has been laser-focused on unraveling the intricate relationship between eating habits and childhood obesity. He has not only investigated the dietary risk factors contributing to this condition (38), but has also probed into the nuanced correlation between breakfast consumption and body weight management (39), as well as the pivotal role of the family environment in shaping eating behaviors (40). Furthermore, Luis has conducted an exhaustive exploration into the link between nutrient intake and adolescent health, with a particular emphasis on the salutary effects of the Mediterranean diet on adolescent health indicators (41, 42). His seminal work is instrumental in unraveling the complexities of obesity, formulating efficacious prevention strategies, and enhancing the well-being of children and adolescents.

Prof. Barry Popkin has the highest H-index of all authors and is also highly cited, which places him at the top of his peer group. His research is widely cited, highlighting his authority in the field and reflecting the far-reaching impact his research has had on the advancement of science. Also, in 2004, Professor B’s research analyzed the diet, activity, and obesity status of adults and children in different countries by reviewing the results of large-scale surveys and nationally representative studies. It proposed the theory of “nutritional transition” (43). This theory provides a critical framework for understanding the evolution of global dietary and physical activity patterns and their impact on public health. In addition to his theoretical contributions, Prof. Popkin has been an active contributor to international collaborations, working with researchers around the world to investigate the complex relationship between eating habits and body weight in children from different regions (44–46). His cross-cultural research has enhanced our understanding of global health issues and provided a scientific basis for developing effective interventions.

It is worth noting that the number of citations for Ludwig and Ebbeling is relatively high, which suggests that some of their research has indeed made essential breakthroughs or is of significant academic value; for example, in 2012, they jointly explored the effects of sugary beverages on adolescent body weight (47). And then, in 2014 and 2015, they jointly explored studies on fast food consumption associated with weight gain (48, 49). Furthermore, the more active authors in the research field, such as the cluster represented by Moreno, Kersting, and Manios, have the broadest range of collaborative research (Figure 5B). In contrast, the other clusters consist mainly of two to three authors, which implies that these authors need more communication and collaboration with other authors in the research field. The above observations show that the three most influential academics are also the most active communicators in their field. This further highlights their role as academic and research leaders, as they are invested in their research topics and actively communicate and collaborate with their peers.

Most papers in the field are published in core journals (Figure 6A). Nutrients is the clear leader in research on eating behaviors related to overweight and obesity in adolescents, with 175 publications and other journals lagging significantly behind. Nutrients has an H-index of 23 and an impact factor of Q1/5.9, demonstrating its authority in the field and its articles’ high quality and impact. However, it is worth noting that “The International Journal of Obesity” has a relatively stable publication volume, and its H-index is 31, representing the most cited frequency and influence of its articles in the academic world. BMC PUBLIC HEALTH,” “OBESITY REVIEWS,” “OBESITY,” and other journals, as the core journals in the field, are equally important in terms of the number of articles and impact factor. In addition, after mid-2010, the number of publications in each journal increased significantly; this trend is even more pronounced between 2019 and 2023, especially in the journal Nutrients and Obesity Reviews, suggesting that the global problem of overweight and obesity, especially among adolescents, has attracted a great deal of attention from researchers due to its increasing prominence.

The journal double citation maps show this study’s distribution across journal fields and the citation relationships between the different journal fields. Figure 6B shows that the fields of MEDICINE/MEDICAL/CLINICAL and PSYCHOLOGY/EDUCATION/ HEALTH have published several studies related to the eating behaviors of adolescents and young adults with overweight/obesity. Figure 6B shows that MEDICINE/MEDICAL/CLINICAL has published several studies related to the eating behaviors of adolescents and young adults with overweight/obesity. While MEDICINE/MEDICAL/CLINICAL research focuses on molecular biology and genetics to advance precision medicine and personalized treatments, PSYCHOLOGY/EDUCATION/ HEALTH research focuses on the eating behaviors of overweight/obese adolescents and young adults, going beyond the physiological dimension to include multiple factors such as psychology and education.

### 4.2 Analysis of research hotspots and trends

The amalgamation of reference co-citation analysis and high-frequency keyword analysis has illuminated the research focal points within dietary behavior studies in overweight and obese children and adolescents. These focal points predominantly revolve around: Epidemiology and Environment factors of obesity; Health risks associated with childhood obesity; key eating habits and interventions for childhood obesity; prevention and treatment of childhood and adolescent obesity.

#### 4.2.1 Epidemiology and environment factors of obesity

##### 4.2.1.1 Global epidemiological trends in childhood overweight and obesity

From 1990 to 2022, obesity among children and adolescents has increased significantly in 188 countries and regions worldwide. In 1990, the global obesity rates for school-age children were 1.7% for girls and 2.1% for boys, respectively. By 2022, these rates had climbed to 6.9 and 9.3%, respectively, highlighting the growing obesity issue (50).

Especially during the COVID-19 pandemic, there was a dramatic increase in eating disorders as a result of work stoppages that limited outdoor physical activity for children and adolescents, altering their lifestyle habits, such as diet and sleep patterns, resulting in weight gain (51, 52). Global consumption of sugar-sweetened beverages (SSBs) has increased, especially in high-income countries, with intake exceeding the recommended daily limit for free sugars. This trend is also increasing in low- and middle-income countries (53, 54). In some countries, adolescents are deriving an increasing proportion of their energy from ultra-processed foods, and additionally, global consumption of fruits and vegetables among adolescents is insufficient (55, 56). Overall, the increasing problem of obesity in children and adolescents is closely related to changes in dietary habits.

##### 4.2.1.2 Environmental factors affecting weight in children and adolescents

The weight status of children and adolescents is significantly influenced by multiple environmental factors that create a complex, interconnected ecosystem that includes the family, community, and school. The home environment significantly affects the type of food available to children. Low-income families, for instance, may favor fast food due to cost considerations (57). Home-cooked meals help prevent overweight and obesity in children (58). Family meals not only help prevent overweight and obesity but also promote mental health, reduce eating disorders, and foster positive eating habits (59, 60). Conversely, frequent dining out may reduce children’s opportunities to learn cooking skills and establish healthy eating habits (61).

Healthy neighborhood environments promote increased fruit and vegetable intake in children and adolescents (62). A community’s diverse range of food outlets, including supermarkets, fast food restaurants, and convenience stores, is often linked to higher BMI (63, 64). Recreational environments within a community, such as park density (65), park size (66), and more sports venues (14), influence weight and related behaviors to some extent. However, existing research often focuses on single community characteristics and fails to comprehensively understand the community’s overall impact on diet and activity.

Children spend most of their time at school, where a significant portion of their energy intake and expenditure occurs (67). Food environments near schools have been linked to varying weight statuses in adolescents (68). For instance, more convenience stores and candy stores near schools have been linked to increased body weight in school-aged children (69). In addition, peer groups at school significant influence on the dietary choices of children and adolescents (70), and peer influence can be both positive and negative. For example, when groups of friends share healthy food choices, they help reduce unhealthy eating behaviors during adolescence (68).

In summary, family, community, and school settings play crucial roles in managing the weight of children and adolescents. Addressing these environmental factors and implementing comprehensive interventions is crucial to prevent and control childhood obesity effectively.

#### 4.2.2 Health risks associated with childhood obesity

Childhood obesity is a growing global health problem and the starting point of a complex metabolic disorder associated with serious health risks.

Insulin resistance (IR), central to obesity-induced metabolic disorders, is crucial in the development of complications such as metabolism-associated fatty liver disease (MAFLD), type 2 diabetes mellitus, and cardiovascular disease. These complications involve complex metabolic pathways (71). Studies show that unhealthy dietary habits, such as high calorie intake, excessive fructose consumption, and irregular eating patterns, can induce insulin resistance, accelerating the development of these diseases (72).

Childhood obesity and its metabolic risk factors significantly increase the risk of atherosclerotic cardiovascular disease (ASCVD) and associated mortality (73, 74). Childhood food insecurity is linked to CVD risk factors. Diets high in saturated fats increase low-density lipoprotein cholesterol (LDL-C) and triglycerides while decreasing high-density lipoproteins (HDL), raising the risk of ASCVD (75). Substituting saturated fatty acids with refined carbohydrates can exacerbate atherosclerotic dyslipidemia caused by obesity (76). Cross-sectional and longitudinal studies show that increased sugar intake is associated with higher triglyceride levels and lower HDL levels (77, 78).

MAFLD, a liver disease linked to the metabolic syndrome, has been renamed from NAFLD (79). In children, obesity contributes to MAFLD development through various mechanisms, including excessive consumption of ultra-processed foods, fats, and added sweeteners like fructose (80, 81). High-calorie diets rich in fat and fructose may promote MAFLD by favoring IR or increasing central obesity (82).

To mitigate the risk of MAFLD, type 2 diabetes, and cardiovascular disease, enhancing children’s diets by limiting high-calorie, high-fat, and high-sugar foods while advocating for healthy lifestyles is imperative to diminish these enduring health hazards.

#### 4.2.3 Key eating habits and interventions for childhood obesity

Good dietary habits are crucial for maintaining good health and managing weight. Regular consumption of energy-dense snacks, mainly those low in nutritional value but high in energy, has been linked to an increased risk of overweight and abdominal obesity in children (83, 84). Certain behaviors, such as screen time (TV, cell phone) during meals, have been associated with a higher likelihood of children consuming unhealthy foods like sugary beverages, chips, and cookies (85).

Sugar-sweetened beverages are a significant source of caloric intake for overweight and obese children. Consuming liquid calories often results in less satiety, leading to an inadequate compensatory reduction in dietary energy intake (86), thereby increasing overall energy consumption. Notably, this additional energy intake is not offset by reducing energy intake in subsequent meals (87), suggesting that consuming sugary beverages may contribute to a long-term energy surplus. Furthermore, children who consumed calorie-free beverages had lower BMIz scores and body fat than those who consumed sugary beverages (88), indicating that the calories in sugary beverages tend to exceed typical calorie consumption, thereby promoting weight gain.

Skipping breakfast has been associated with an increased risk of obesity or overweight in children (89). Additionally, a low intake of fruits and vegetables is a risk factor for obesity. Fruits and vegetables have high water and fiber content, which effectively lowers the energy density of the diet and enhances satiety. This helps to reduce overall energy intake and consequently lowers the risk of obesity (90).

Eating attitudes and behavioral disorders tend to be more pronounced in obese children compared to their normal-weight peers, and these issues may be worsened by additional physical health problems and psychosocial stressors (91). These disorders are often linked to a lack of dietary knowledge. Research indicates that adolescents with moderate dietary knowledge are less likely to be overweight or obese (11). Moreover, the dietary knowledge and attitudes of parents play a crucial role in shaping their children’s eating behaviors (92). The above studies suggest that improving children’s and parents’ understanding of healthy eating can effectively prevent and manage childhood obesity.

Weight perception, which encompasses an individual’s assessment of their body image and their perception of weight and appearance, is essential for children and adolescents. When children lack proper weight perception, they may not recognize that they are overweight or obese, a cognitive bias that is more common among students (93). Living in an environment where obesity is prevalent can further exacerbate these misperceptions of weight status (94). While weight perception itself does not directly determine body weight, children who perceive themselves as overweight or obese tend to be more knowledgeable about obesity-related information and are often more motivated to lose weight. However, they may also be more likely to adopt unhealthy lifestyles to manage their weight (95).

In summary, addressing childhood obesity requires a multifaceted approach. Efforts should improve children’s eating environments and behaviors, enhance parents’ and children’s dietary knowledge, and increase awareness about weight perception. This necessitates developing and implementing comprehensive interventions through collaborative efforts across various disciplines.

#### 4.2.4 Prevention and treatment of childhood and adolescent obesity

##### 4.2.4.1 Prevention of obesity in children and adolescents

Once obesity occurs in children, achieving effective and lasting results through lifestyle changes alone can be challenging (96). Thus, timely prevention of childhood obesity is crucial. Common strategies for preventing childhood obesity include reducing overall food consumption or energy intake, increasing the consumption or preference for fruits and vegetables, and reducing sugar intake or preference (97).

A healthy dietary pattern and strategy should be based on individual preferences and circumstances, family environment, and available support. For example, the Mediterranean diet, on the one hand, the MedDiet diet avoids the intake of ultra-processed foods, which tend to lead to higher calorie intake (98), and on the other hand, it recommends plant-based foods that are low in energy density and large in body mass, which increase satiety while reducing energy intake.

Developing proper dietary knowledge and attitudes is essential. An individual’s dietary knowledge significantly influences their dietary choices, and the dietary knowledge of parents, especially mothers, also impacts their children’s dietary choices (99). Improving dietary knowledge among children and adolescents can reduce the risk of overweight and obesity (12). Therefore, dual interventions at school and home are crucial strategies for promoting healthy dietary habits and preventing childhood obesity.

##### 4.2.4.2 Intervening in the eating behaviors of overweight/obese children and adolescents in the environment

Schools are critical sites for preventing childhood obesity. Eating habits of elementary school children can be positively influenced by optimizing food offerings in school cafeterias and kiosks, increasing healthy food options, and implementing nutrition education curricula focused on balanced diets and healthy nutritional behaviors (100, 101). Encouraging parental involvement and providing healthy eating education through newsletters, workshops, and parent-teacher conferences are also effective strategies (102). A school- and home-based prevention trial using the CHIRPY DRAGON approach proved significant and cost-effective in reducing BMI z-scores in Chinese primary school children. While school-based interventions have a modest positive impact on childhood obesity in the short term, their long-term effects appear limited (103).

Community-based interventions (CBIs) have the potential to reduce obesity rates in populations through multi-channel education, utilization of community resources, provision of professional support, policy, and environmental changes, and specific interventions in diet and lifestyle habits (104–106). However, these interventions face challenges such as low effectiveness, high complexity, resource and continuity issues, individual differences and variable participation, and difficulties in evaluation and monitoring.

Family-based interventions are crucial for preventing and treating childhood obesity. Family-based treatment (FBT), which employs a cognitive behavioral therapy (CBT) approach to address diet and physical activity through behavioral change strategies, is recognized as the gold standard in treating childhood obesity (107). FBT not only helps children and parents achieve clinically significant weight loss but also supports maintaining a healthy weight into adulthood (108). Furthermore, FBT is more cost-effective than individual treatment and is recommended for screening and referring children with obesity. FBT can be combined with socially facilitated maintenance (SFM) to strengthen social networks and improve body image, further supporting the maintenance of healthy behaviors. Despite concerns that FBT may increase the risk of eating disorders, available evidence suggests that evidence-based weight loss programs reduce eating disorder pathology (109).

Although school, community, and family-based interventions have shown positive results in preventing and treating childhood obesity—particularly in terms of short-term cost-effectiveness and reductions in BMI z-scores—further research is needed on their long-term effects and sustainability. Future research should focus on detailed tracking of resource utilization, evaluation of long-term outcomes, and consideration of broader intervention impacts. This approach aims to optimize strategies and address existing challenges, such as insufficient significant effects, resource limitations, persistence issues, and individual differences.

##### 4.2.4.3 Dietary clinical management of overweight and obesity in children and adolescents

Clinical trials for pediatric populations have commonly employed targeted behavioral interventions, such as dietary modifications and increased physical activity, to address the imbalance between calorie intake and expenditure. These interventions are often supplemented by pharmacological or surgical treatments when necessary (8). However, there is currently no consensus on the optimal dietary regimen for treating childhood obesity. Given the unclear impact of adult eating patterns on long-term growth and nutritional status in children (110), clinical interventions—especially dietary interventions—should be conducted under the close supervision of a clinician to mitigate the risk of eating disorders (111). Clinically significant weight loss in children has been associated with younger age, mild to moderate obesity, and reduced consumption of sugary beverages (112). Additionally, low-calorie and no-calorie sugar-sweetened beverages can be effective alternatives to sugary beverages, producing weight loss effects similar to those of water (113).

Recent studies have indicated that very high doses of vitamin D (>4,000 IU/day) supplementation can effectively reduce inflammation levels and improve insulin sensitivity in overweight/obese children and adolescents (114). Moreover, the use of probiotics and synbiotics has been proposed to aid in fat loss among overweight and obese individuals (115). Probiotics have demonstrated potential in modulating levels of HDL-C, LDL-C, lipocalin, leptin, and TNF-*α* in overweight or obese children, potentially alleviating metabolic symptoms associated with obesity (116). However, there are notable individual differences in the effects of prebiotics on short-chain fatty acids (SCFA) produced by gut microbes in obese adolescents (117), which could account for their variable effects in childhood obesity.

Mobile health (mHealth) has emerged as a promising approach for managing overweight and obese children and adolescents, mainly by providing convenient remote access for those who cannot regularly attend medical appointments. Using mobile devices for self-monitoring has proven effective (97). Smartphone apps have shown positive results in improving obesity-related behaviors, such as reducing the intake of sweet and salty foods, sweetened beverages, and limiting screen time (118). Additionally, integrating nutritional education content into mobile games can effectively guide children toward making healthier food choices (119). However, there is currently a lack of evidence demonstrating that mHealth devices are directly associated with a decrease in BMI, and further investigation is necessary to establish their efficacy in this regard.

In summary, future research should explore more precise nutritional intervention strategies that incorporate genetic, metabolic, and lifestyle factors to effectively manage childhood obesity. By considering these individual differences, tailored interventions that optimize outcomes for each child may be possible. Further investigation into the long-term effects of these interventions, including high-dose vitamin D supplementation, probiotics, and mHealth tools, is also essential to establish their efficacy and safety in managing childhood obesity.

### 4.3 Limitations

The present study, while significant, carries several limitations. First, it used only one database, the WoSCC, excluding others like Google Scholar and PubMed, which could provide a broader data scope. Second, the study focused on reviews and articles, omitting other scholarly contributions, such as books, which may offer valuable insights. This limitation might exclude pivotal studies conducted in different formats, restricting the conclusions drawn. Additionally, the research hotspots were based on a selection of 2,142 papers, potentially overlooking emerging or less-investigated topics crucial for understanding obesity. Future research should refine this approach by including diverse databases, exploring interdisciplinary and cross-sectional studies, and incorporating various research types to explore the subject comprehensively.

## 5 Conclusion

This research provides a comprehensive overview of global trends and key areas in studying dietary behaviors among overweight/obese children and adolescents. It offers a detailed summary of recent advancements, emphasizing this field’s critical principles and practices. By exploring these developments, the study highlights the growing importance of this research within global healthcare and suggests pathways for future research and applications.

## Data Availability

The original contributions presented in the study are included in the article/supplementary material, further inquiries can be directed to the corresponding author.
